# Supplementary material for: Longitudinal Association of Universal Screening and Treatment for Major Depressive Disorder with Survival in Cancer Patients
Source: J Pers Med. 2022 Jul 26;12(8):1213. doi: 10.3390/jpm12081213 (PMC9331985; doi:10.3390/jpm12081213)
Supplement: Supplementary file 1 [file jpm-12-01213-s001.zip › jpm-1739665-supplementary.pdf]

Journal of *Personalized Medicine*

MDPI

Supplementary Materials

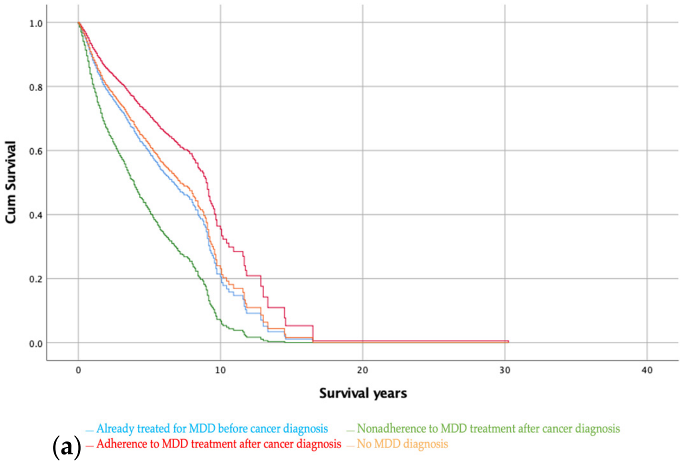

(a)

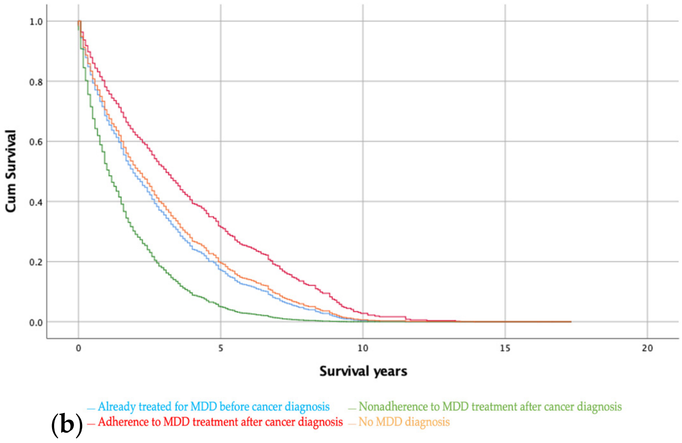

(b)

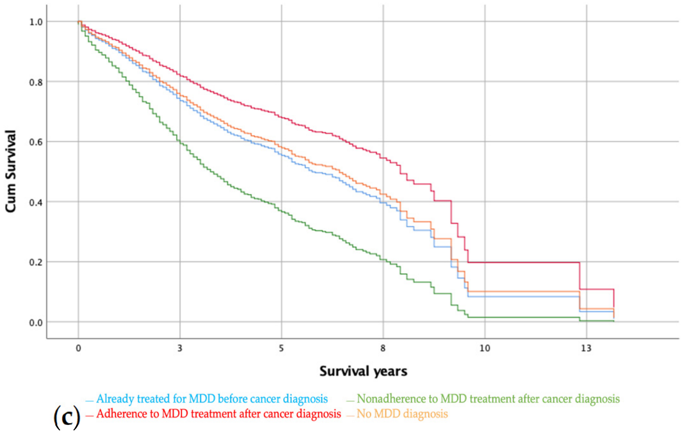

(c)

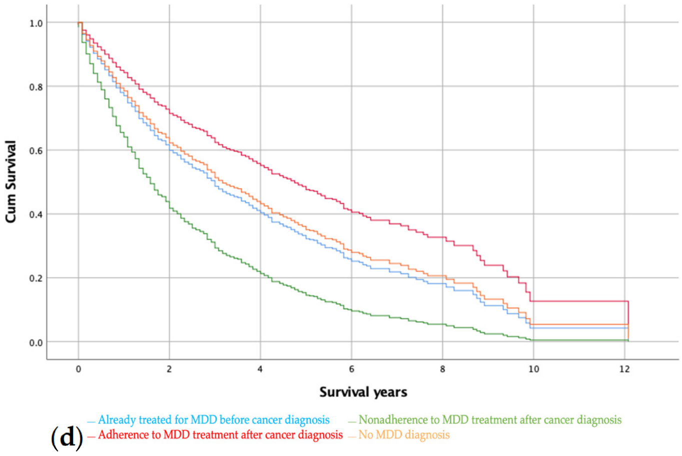

(d)

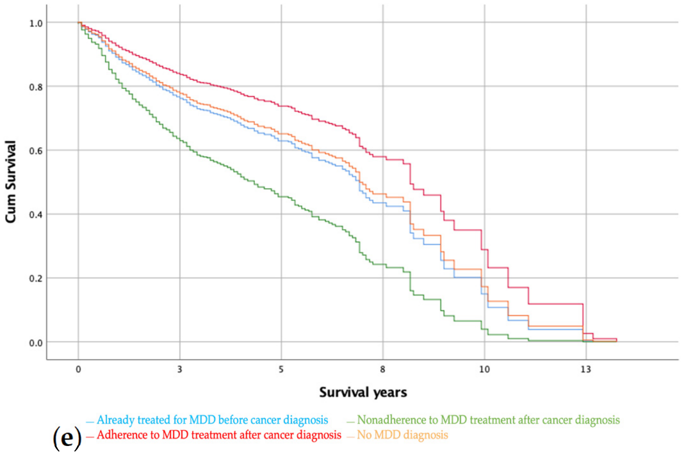

(e)

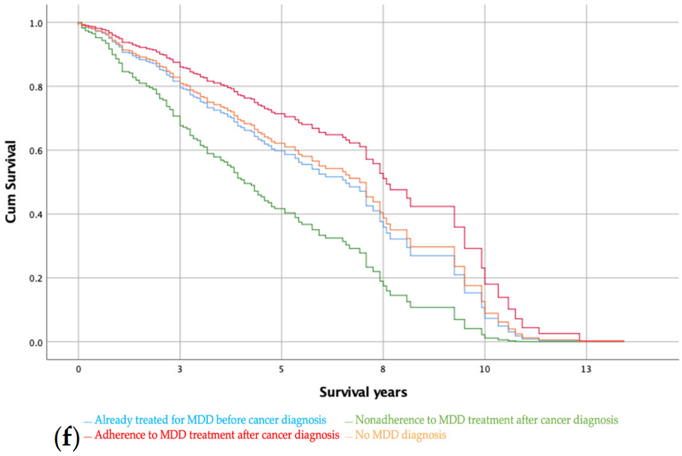

(f)

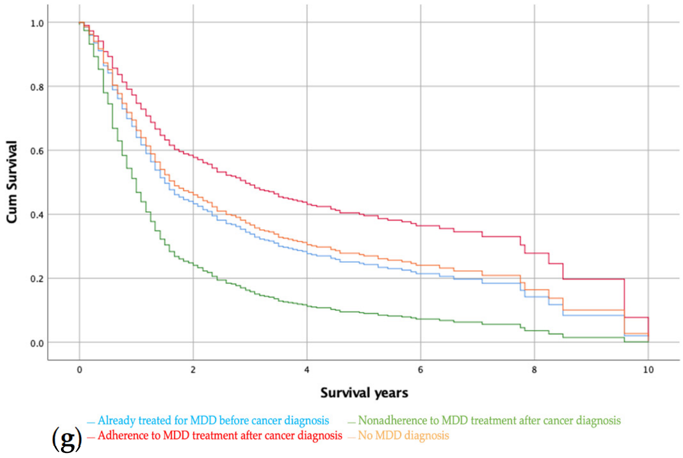
Cum Survival
Survival years
Already treated for MDD before cancer diagnosis
Nonadherence to MDD treatment after cancer diagnosis
Adherence to MDD treatment after cancer diagnosis
No MDD diagnosis
(g)

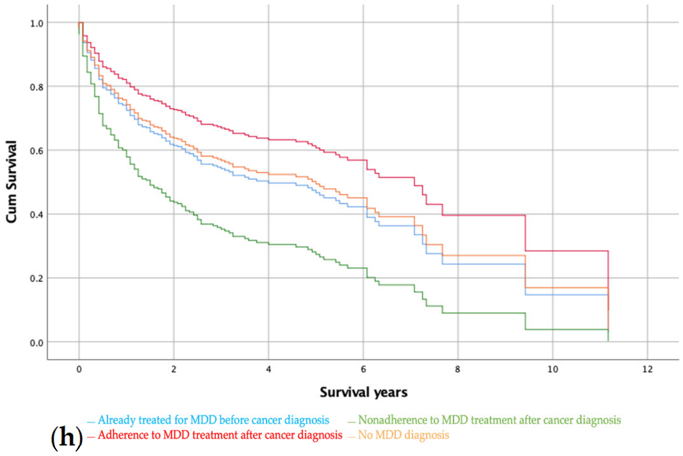
Cum Survival
Survival years
Already treated for MDD before cancer diagnosis
Nonadherence to MDD treatment after cancer diagnosis
Adherence to MDD treatment after cancer diagnosis
No MDD diagnosis
(h)

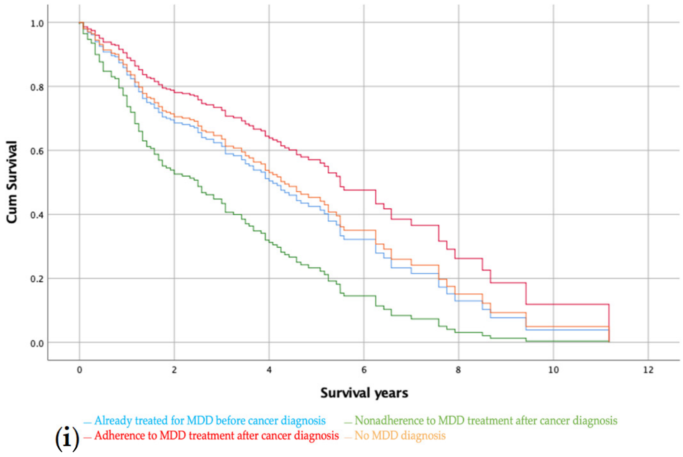
Cum Survival
Survival years
Already treated for MDD before cancer diagnosis
Nonadherence to MDD treatment after cancer diagnosis
Adherence to MDD treatment after cancer diagnosis
No MDD diagnosis
(i)

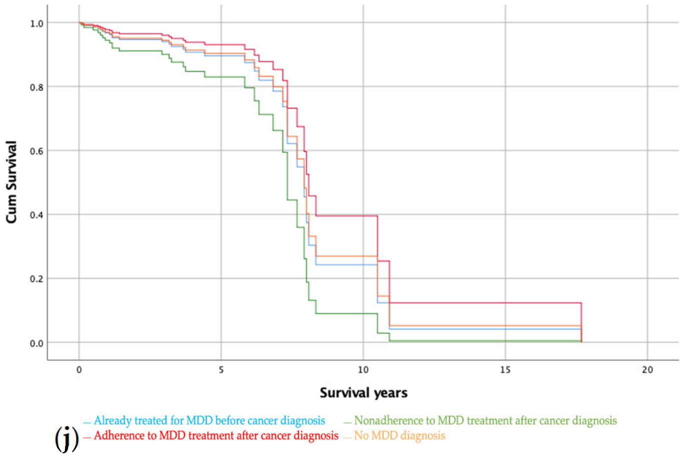
Cum Survival
Survival years
Already treated for MDD before cancer diagnosis
Nonadherence to MDD treatment after cancer diagnosis
Adherence to MDD treatment after cancer diagnosis
No MDD diagnosis
(j)

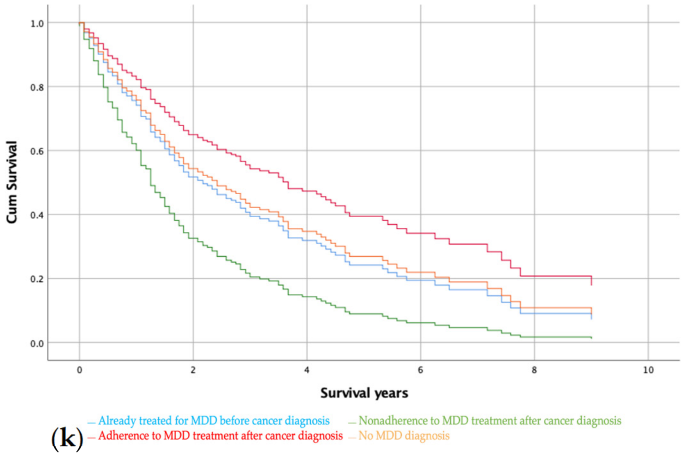
Cum Survival
Survival years
Already treated for MDD before cancer diagnosis
Nonadherence to MDD treatment after cancer diagnosis
Adherence to MDD treatment after cancer diagnosis
No MDD diagnosis
(k)

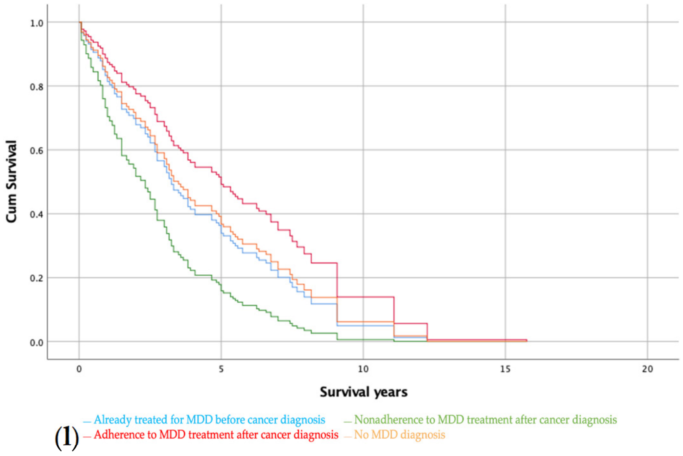
Cum Survival
Survival years
Already treated for MDD before cancer diagnosis
Nonadherence to MDD treatment after cancer diagnosis
Adherence to MDD treatment after cancer diagnosis
No MDD diagnosis
(l)

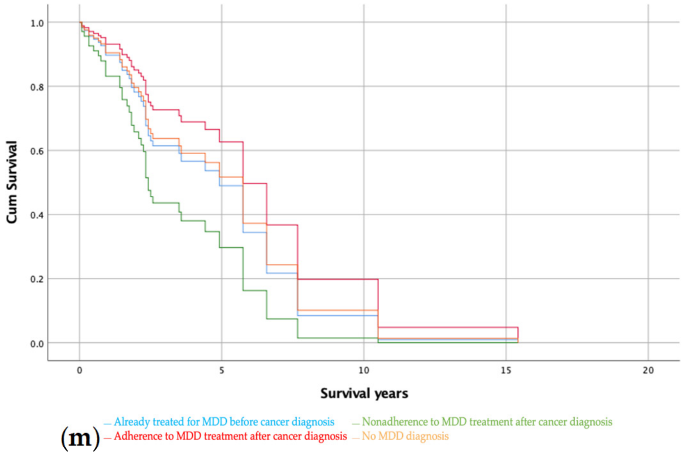
Cum Survival
Survival years
Already treated for MDD before cancer diagnosis
Nonadherence to MDD treatment after cancer diagnosis
Adherence to MDD treatment after cancer diagnosis
No MDD diagnosis
(m)

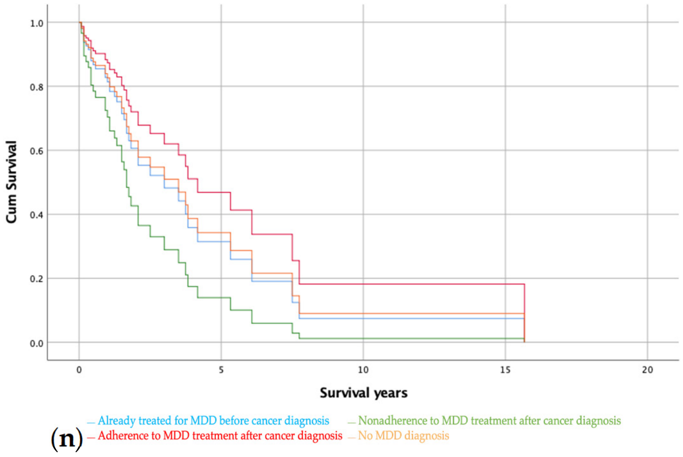
Cum Survival
Survival years
Already treated for MDD before cancer diagnosis
Nonadherence to MDD treatment after cancer diagnosis
Adherence to MDD treatment after cancer diagnosis
No MDD diagnosis
(n)

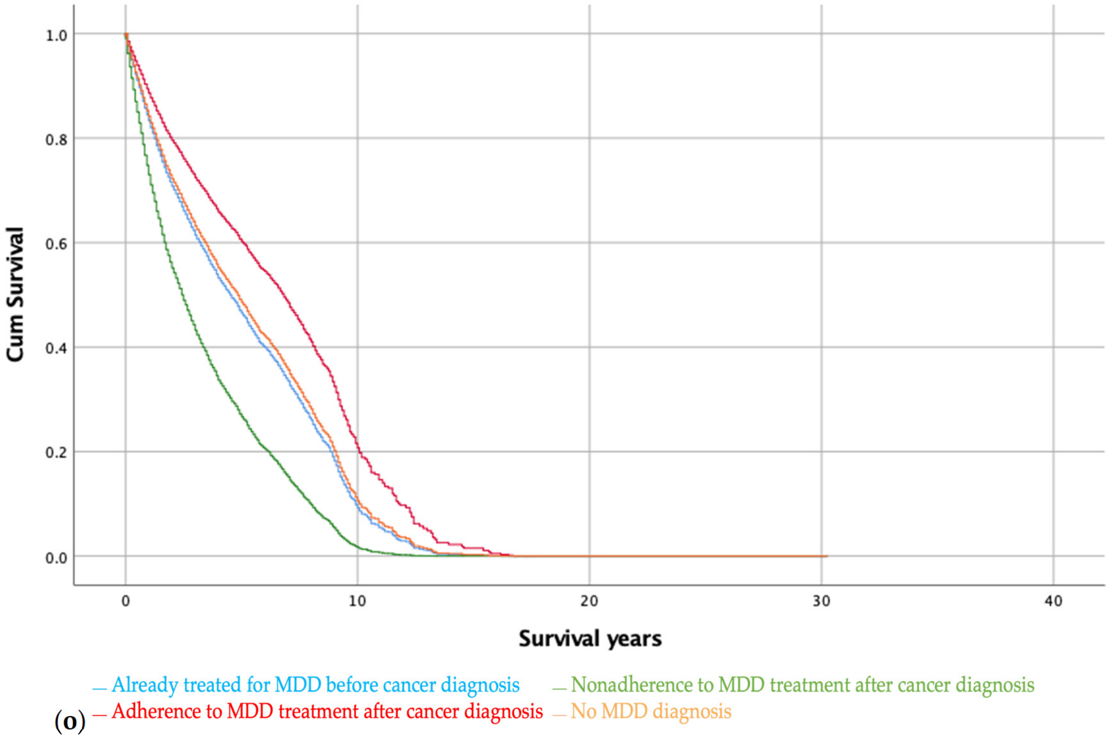

(**o**)

Figure S1. Survival functions of the Cox proportional hazards regression model stratified by (**a**) head/neck, (**b**) liver/pancreas, (**c**) colorectal, (**d**) lung, (**e**) genitourinary, (**f**) breast, (**g**) esophagus, (**h**) hematological, (**i**) gynecological, (**j**) thyroid, (**k**) gastrointestinal, (**l**) bladder, (**m**) skin, (**n**) brain/nerves, and (**o**) overall, after adjustment for sex, age at cancer diagnosis, age at screening for MDD, and cancer stage. MDD: major depressive disorder.
